# Molecular Characteristics of Methicillin-Resistant and Susceptible *Staphylococcus aureus* from Pediatric Patients in Eastern China

**DOI:** 10.3390/pathogens12040549

**Published:** 2023-04-02

**Authors:** Yuxuan Zhou, Shuyang Yu, Chenjun Su, Shengqi Gao, Guilai Jiang, Zhemin Zhou, Heng Li

**Affiliations:** Pasteurien College, Suzhou Medical College of Soochow University, Suzhou 215123, Chinazmzhou@suda.edu.cn (Z.Z.)

**Keywords:** *Staphylococcus aureus*, children, MLST type, antibiotic resistance, virulent genes

## Abstract

*Staphylococcus aureus* is an opportunistic pathogen that causes invasive infections in humans. In recent years, increasing studies have focused on the prevalence of *S. aureus* infections in adults; however, the epidemiology and molecular characteristics of *S. aureus* from Chinese pediatric patients remain unknown. The present study examined the population structure, antimicrobial resistance, and virulent factors of methicillin-resistant and -susceptible *S. aureus* isolated from Chinese pediatric patients from one medical center in eastern China. A total of 81 cases were screened with positive *S. aureus* infections among 864 pediatric patients between 2016 and 2022 in eastern China. Molecular analysis showed that ST22 (28.4%) and ST59 (13.6%) were the most typical strains, and associations between different clonal complex (CC) types/serotype types (ST) and the age of pediatric patients were observed in this study. CC398 was the predominant type in neonates under 1 month of age, while CC22 was mainly found in term-infant (under 1 year of age) and toddlers (over 1 year of age). Additionally, 17 *S. aureus* isolates were resistant to at least three antimicrobials and majority of them belonged to CC59. The *blaZ* gene was found in 59 isolates and *mecA* gene was present in 26 strains identified as methicillin-resistant. Numerous virulent factors were detected in *S. aureus* isolated from present pediatric patients. Remarkably, *luk*F-PV and *luk*S-PV were dominantly carried by CC22, *tsst*-1 genes were detected in CC188, CC7, and CC15, while exfoliative toxin genes were found only in CC121. Only 41.98% of the *S. aureus* isolates possessed *scn* gene, indicating that the sources of infections in pediatric patients may include both human-to-human transmissions as well as environmental and nosocomial infections. Together, the present study provided a phylogenetic and genotypic comparison of *S. aureus* from Chinese pediatric patients in Suzhou city. Our results suggested that the colonization of multi-drug resistant isolates of *S. aureus* may raise concern among pediatric patients, at least from the present medical center in eastern China.

## 1. Introduction

The methicillin-resistant and sensitive *Staphylococcus aureus* (MRSA/MSSA) is the most prevalent invasive bacterial pathogen that infects children globally [1,2]. According to current epidemiological studies in both adults and children, the incidence of MRSA is declining globally, while the frequency of invasive community-associated MSSA infection has remained stable or even increased [3,4]. Previous studies in the United States and China revealed that the median age of children with invasive *S. aureus* infections was 6.3 to 8.7 years and that MSSA caused 79% of cases in the United States and 36.0% of *S. aureus* infections in Chinese pediatric patients [5,6,7,8].

*Staphylococcus aureus* in children may cause a range of infections such as soft tissue infection (SSTIs), pneumonia, primary bacteremia, otitis media, septic arthritis, and others [9]. According to a recent study, 84.2% of *S. aureus* strains in the stool of Chinese children were resistant to penicillin, with *blaZ* being the main antimicrobial gene of these intestinal strains [10,11]. Additionally, *S. aureus* contains several genes encoding virulent factors, including enterotoxins (SE) *sea* to *see*, *seg* to *sei*, *ser* to *set*, exfoliative toxins (*eta*, *etb*), toxic shock syndrome toxin (*tsst*-1), Panton–Valentine leukocidin (*luk*S/F-PV), staphylococcal complement inhibitor (*scn*), and hemolysins (*hly/hla*, *hlb*, *hld*, *hlgA*, *hlgB*, *hlgC*) [12,13,14,15,16,17]. Previous studies have reported numerous antimicrobial resistance (AMR) and virulent factors (VF) of *S. aureus* detected in Chinese adults [18]. Statistically, 92.2% of the MSSA isolates were penicillin-resistant, whereas 45.3% were erythromycin-resistant. ST398 and ST15 were the most common enteric MSSA isolates in these adult individuals [18]. However, the molecular features of *S. aureus* infections in children in eastern China over the past five years remain to be investigated.

To characterize MRSA and MSSA in eastern China from 2016 to 2022, a combined bioinformatics approach was employed to study the relatedness of *S. aureus* isolated from pediatric patients. In addition, we examined genotypic antimicrobial resistance profiles and virulent factor possession for a comprehensive assessment of the potential risks associated with the presence of MRSA and MSSA in children.

## 2. Materials and Methods

### 2.1. Ethics Approval

The experiment was strictly conducted according to the Guide for the Care and Use from the Research Ethics Committee of Soochow University (20221201). All procedures involving human participants were performed in accordance with ethical standards. Patients’ parents were given informed consent to participate in the study.

### 2.2. Description of the Medical Center

The current study was carried out at the Children’s Hospital Affiliated with Soochow University in eastern China (120°62′ E, 31°32′ N). A tertiary hospital was established at this medical facility in 1959. It primarily provides services to the cities of Taizhou, Changzhou, Nantong, and other places. The hospital witnessed 2,340,727 outpatient and emergency visits in total in 2022. It has a strong representation and is totally in charge of diagnosing, treating, and caring for children in Suzhou and the surrounding regions in eastern China.

### 2.3. Clinic Data of Pediatric Patients

We initially analyzed the clinic data of 864 pediatric patients in the present medical center from 2016 to 2022. A number of 81 cases were screened with positive *S. aureus* infections. For each patient, we collected clinical metadata of age, gender, underlying diseases, and previous hospitalization. None of the 81 pediatric patients had long-term hospitalization history and they developed symptoms such as acute infection before being admitted to the hospital for treatment. Detailed clinic data of pediatric patients were described in the Results section and Supplementary Table S1. 

### 2.4. Diagnosis of Staphylococcus aureus Infection

The diagnosis was obtained by trained clinical staff from the Department of Infectious Diseases in the present medical center according to the guidelines for the treatment of MRSA infection in adults and children issued by the Infectious Diseases Society of America (IDSA) [19] and strategies for the management of methicillin-resistant *Staphylococcus aureus* infections by an expert panel of the Chinese Medical Association (https://www.cma.org.cn, accessed on 1 October 2016). Taking acute otitis externa as an example, following skin disinfection with 75% ethanol, clinical staff used a sterile cotton swab to collect purulent secretions in the child’s ear canal. If the tympanic membrane has not been ruptured and perforated, then puncture it to absorb the pus and place it in a sterile tube for examination. Quality control strains were employed to eliminate contamination by miscellaneous bacteria including *Staphylococcus aureus* (ATCC29213), *Streptococcus pneumoniae* (ATCCA9619), *Escherichia coli* (ATCC25922), *Pseudomonas aeruginosa* (ATCC27853), and *Candida albicans* (ATCCl0231). Only children diagnosed with *S. aureus* single or mixed infection by the Department of Infections were included in this study. The obtained *S. aureus* strains were confirmed by 16s rRNA sequencing and MALDI-TOF MS (BioMérieux, Craponne, France).

### 2.5. Staphylococcus aureus Strains

A total of 81 *S. aureus* isolates were collected from pediatric patients between 2016 and 2022 in Suzhou, China. Among the 81 isolates, 26 isolates were identified as MRSA, with the majority of the strains collected from otitis externa (n = 23), otitis media (n = 11), and soft tissue infection (n = 9).

### 2.6. Antimicrobial Susceptibility Testing

Confirmed *S. aureus* strains were sent for antimicrobial susceptibility test by disk diffusion (Oxoid) according to the guideline of the Clinical and Laboratory Standards Institute (CLSI 2022). The antimicrobial compounds included ampicillin (10 μg), cefoxitin (30 μg), ceftazidime (30 μg), cefuroxime (30 μg), chloramphenicol (30 μg), clindamycin (2 μg), erythromycin (15 μg), gentamicin (10 μg), linezolid (30 μg), penicillin (10 units), rifampicin (5 μg), and tetracycline (30 μg), with *S. aureus* ATCC 25923 enrolled as the control strain. Cefoxitin was used to confirm potential methicillin-resistant *S. aureus* (MRSA).

### 2.7. Whole Genome Sequencing

Isolates were cultured for 24 h at 37 °C in Tryptone Soya Broth (AOBOX 02-049, Beijing, China), and genomic DNA was extracted and purified using a HiPure Bacterial DNA Kit (D3146, Meiji Biotechnology Co., Ltd., Guangzhou, China). The extracted DNA was tested for quality using a Nanodrop ND-1000 spectrophotometer (Nanodrop Technologies, Wilmington, DE, USA) and a 1.0% (*w*/*v*) agarose gel. Purified DNA was whole genome sequenced via Illumina’s NextSeq 500 on the platform of Honsunbio company (Shanghai, China). EToKi v1.0 was used to generate genome assemblies, and the quality was assessed using QUAST v2.3 [20,21]. The raw sequencing reads were submitted to the China National GenBank under the link https://bigd.big.ac.cn/gsa/browse/CRA009709, accessed on 7 February 2023. 

### 2.8. Analysis of MLST and Clone Complexes

The Illumina read files were submitted to MLST 2.0 multilocus sequence typing, and eBURST v3 analysis was performed to categorize STs into clonal complexes (CCs) [22,23]. CSI Phylogeny v1.4 was used to create a single nucleotide polymorphism (SNP) tree for all isolates by matching other genomes onto a reference genomic sequence from *S. aureus* ATCC 25923 on the Center for Genomic Epidemiology [24].

### 2.9. Identification of Antimicrobial Resistance Genes and Virulent Factors

The online tools ResFinder, MobileElementFinder, and VFDB were used to screen for antimicrobial-resistant genes and virulent factors [25,26,27]. Alignments with a minimum of 60% nucleotide identity were retained in all three algorithms. Grapetree and ITOL were used to depict the genotypic data [28,29]. GraphPad Prism 7 was used to create the violin graph, and statistical significance was determined using One-way ANOVA with *p* < 0.05.

## 3. Results and Discussion

### 3.1. Sequence Types and Clonal Complex of S. aureus from Chinese Pediatric Patients

A total of 81 *S. aureus* were isolated from pediatric patients in this medical center between 2016 and 2022. Among them, ten strains were isolated from neonates under one month old, and 28 strains were coming from term infants under one year old. The remaining 43 strains were all isolated from the toddler age group (12 of them were isolated from the 2–4 years old subgroup, 15 were from the 5–7 years old subgroup, and the rest 16 isolates were from the 8–10 years old subgroup). 

**Figure 1:**
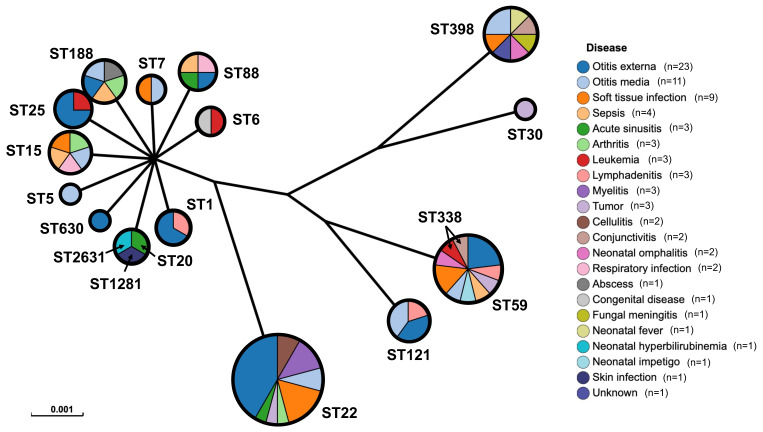
Sequence types of *S. aureus* from Chinese pediatric patients. *Staphylococcus aureus* strains were isolated from otitis externa(n = 23), otitis media(n = 11), soft tissue infections (n = 9), and other diseases, of which ST22 (28.4%) and ST59 (13.6%) were the most typical strains.

The diagnosis of infections was obtained by trained clinical staff in the present medical center. Most strains were isolated from the infection of acute/chronic otitis externa (n = 20/3), acute/chronic otitis media (n = 7/4), soft tissue infections (n = 9), sepsis (n = 4), acute sinusitis (n = 3), arthritis (n = 3), cellulitis (n = 2), conjunctivitis (n = 2) and other infections, of which 26 strains were identified as MRSA (Figure 1). However, several strains were isolated from a mix of infections such as leukemia (n = 3), tumor (n = 3), congenital disease (n = 1), fungal meningitis (n = 1), neonatal hyperbilirubinemia (n = 1) and other unknown infections. Detailed backgrounds on the range of ages and infections were described in Supplementary Tables S1 and S2. 

Notably, 34 strains were isolated from infection of acute/chronic otitis externa and otitis media. Acute otitis media (AOM) is rarely associated with *S. aureus* in adults [30]. However, a previous study reported that *S. aureus* was the second most common cause of AOM in children in Liuzhou, Southern China [31]. Most of the AOM-associated *S. aureus* were MDR strains with a high carriage rate of *erm*A and *erm*C genes. This may be due to the weak immunity in pediatric patients, which makes *S. aureus* infection more frequent in AOM cases [32].

The MLST typing of the 81 *S. aureus* genomes revealed a total of 18 unique ST types, of which ST22 (28.4%) and ST59 (13.6%) were the most typical strains. MSSA isolates (n = 78) were detected in the majority of the ST types except for ST30 and ST338, and in these Chinese pediatric patients, MRSA isolates (n = 14) were found in ST6, ST22, ST30, ST59, ST88, ST121, ST338, and ST398. A total of 17 clonal complex types were identified according to eBURST V3, namely CC1, CC5, CC6, CC7, CC8, CC15, CC20, CC22, CC25, CC30, CC59, CC88, CC121, CC188, CC338, CC398, and CC1281 (Table 1, Supplementary Table S1).

ST22 was most prevalent, originating from otitis externa (n = 9), soft tissue infection (n = 4), myelitis (n = 3), and other pediatric diseases (n = 7). Additionally, ST59 and ST398 were the sources of various infections in this study, indicating the diversity of *S. aureus* infections in these pediatric patients.

**Figure 2:**
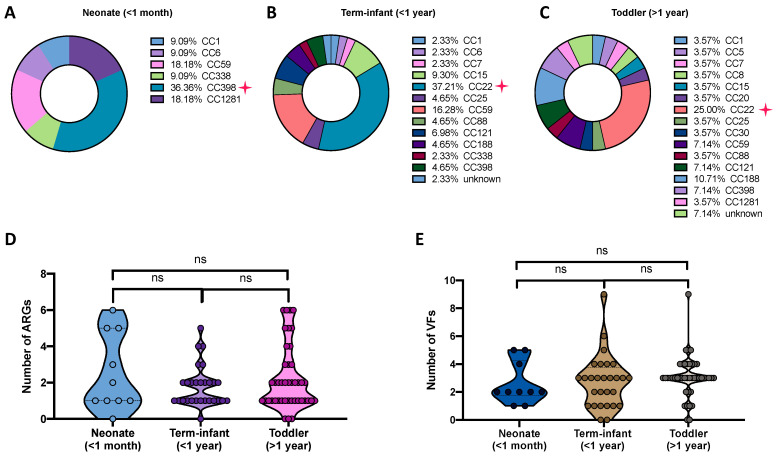
Distribution of clonal complex types among Chinese pediatric patients. (**A**–**C**) CC398 was the predominant type in neonates under 1 month of age, while CC22 was mainly found in term-infant (under 1 year of age) and toddlers (over 1 year of age). (**D**,**E**) No significance of ARGs and VFs were observed between these years group of pediatric patients (*p* > 0.05).

Another interesting finding of this study is the observed association between different CC types/ST and the age of pediatric patients. Thus, CC398 was the predominant type in neonates under 1 month of age (36.36%), while CC22 was mainly found in term-infants (under 1 year of age, 37.21%) and toddlers (over 1 year of age, 25.00%). Only six ST types are detected in neonates, but ST types gradually diversified with age, with more than 15 ST types recorded in toddlers aged 1 to 9 years (Figure 2).

Literally, *S. aureus* CC398 has been identified as the most predominant clonal complex worldwide [33]. Since the first report of CC398 colonization in French swine farms in the late 1990s, this kind has expanded globally, with human and animal infections occurring in Europe and East Asian countries [34]. CC398 is one of the most common *S. aureus* lineages in China, with a prevalence of 5.5% to 26.6%, which shows a lower infection rate compared to our findings among these pediatric patients [35,36,37]. 

Previous studies have reported the wide spread of CC22 in China [38]. A higher frequency of *pvl* and toxin encoding *tsst*-1 for toxic shock syndrome was observed in Chinese CC22 isolates [38]. However, the high prevalence of CC22 was not the case in Europe, where only 8% of the invasive infection were caused by this type of strain [39]. Nevertheless, CC22 isolates usually contain 8 to 13 virulent factors, especially in the nasal cavity of Chinese pediatric patients [40]. In this study, CC22 is the predominant strain in the full-term and toddler groups. All CC22 isolates carried *luk*F-PV and *luk*S-PV factors, indicating that CC22 is highly pathogenic in China. As a result, prompt and accurate screening for hypervirulent MRSA in pediatric patients is critical.

CC59 isolates are primarily responsible for MSSA infections and are one of the most common clonal complexes among pediatric *S. aureus* isolates in China [41]. A previous study on the prevalence of *S. aureus* in China from 2014 to 2020 revealed that CC59 was the most common clone complex, accounting for 31.2% of CA-MRSA lineages [38]. Most CC59 strains in China lack *pvl* and have a low resistance to conventional antimicrobial agents [38]. In this study, ST59 was found in both MRSA and MSSA strains, although only four isolates were *pvl*-positive, suggesting a low pathogenicity but a high prevalence of hypotoxic *S. aureus* infections in children in eastern China.

### 3.2. Phenotypical and Genotypical Antimicrobial Resistance 

**Figure 3:**
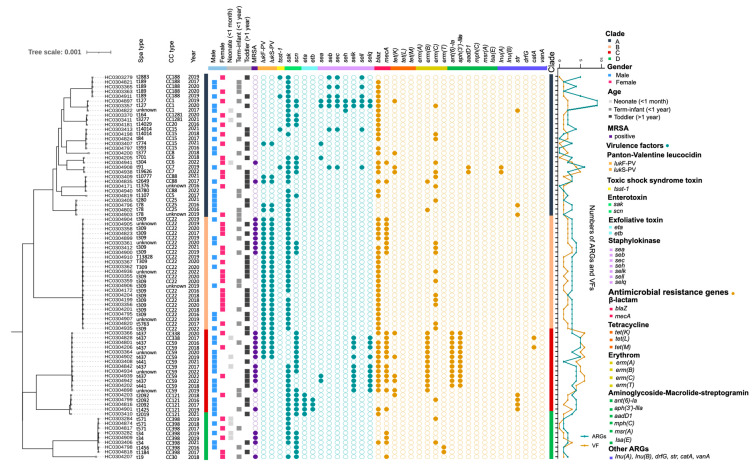
Molecular characteristics of present 81 *S. aureus* strains. The squares colored by trait category represented the presence of age, gender, ARGs, and virulent factors. The line chart showed the total numbers of ARGs and VFs among the 81 *S. aureus* strains.

All 81 *S. aureus* isolates from Chinese pediatric patients were screened for phenotypic antimicrobial-resistant profiles (Figure 3). Among them, 17 isolates were resistant to at least three antimicrobials and the majority of them belonged to CC59 (n = 11). Specifically, the current *S. aureus* isolates were resistant to ampicillin (n = 60), penicillin (n = 67), erythromycin (n = 38), gentamycin (n = 12), cefoxitin (n = 28), and tetracycline (n = 10), whereas a majority were sensitive to ceftazidime, cefuroxime, chloramphenicol, clindamycin, linezolid, and rifampicin, indicating a broad but complex multi-drug resistance of *S. aureus* infections in the current pediatric patients (Table 1).

Given the importance of antimicrobial resistance in pediatric pathogens, we compared the distributions of antimicrobial-resistant genes (ARGs) in 81 *S. aureus* strains from different clades (Figure 3). A total of nine ARGs associated with five classes of antimicrobial agents were present in the isolates from four clades, among which 13.33% (4/30, clade A), 8.33% (2/24, clade B), 72.22% (13/18, clade C), and11.11% (1/9, clade D) of isolates from the corresponding clades were identified as multi-drug resistance (Figure 3). Notably, the *blaZ* gene was found in 59 isolates and the *mecA* gene was present in 26 strains, which were consistent with our phenotypical tests. The *tet*(K) and *erm*(C) genes were distributed widely among CC1 (33%, 33%), CC7 (100%, 50%), CC22 (not detect for *tet*(K), 39.13%), and other types. CC59 carried several unique genes including *erm*(B), *ant*(6)-*la*, and *aph*(3′)*-IIIa.* Finally, we calculated the prevalence of ARGs among these isolates, with clade C having the highest numbers of ARGs including strains of CC59, CC121, and CC338. However, further statics indicated that no significance was observed between these years group among present Chinese pediatric patients (*p* > 0.05, Figure 2).

Ampicillin, tetracycline, penicillin, and erythromycin resistance were commonly found in *S. aureus* isolates from human infections [18]. Similarly, resistance genes to β-lactam antimicrobials, tetracycline, and erythromycin were identified in this study. This might be caused by the common usage of such antimicrobial classes in clinical treatment [42]. Additionally, the *blaZ* gene encodes β-lactamase, which confers penicillin resistance, and this gene may be present on transposons, insertion sequences, plasmids, and other mobile elements [43]. In the current investigation, we discovered that 93.0% of *S. aureus* isolates were resistant to penicillin, which is comparable to a previous report from China that 84.2% of fecal *S. aureus* in children were resistant to penicillin [11]. In contrast, Spanish research found that only 40% of *S. aureus* in neonatal stool was resistant to penicillin [44]. 

On the other hand, all present *S. aureus* isolates tested negative for vancomycin and linezolid, which is consistent with previous isolations from feces from other countries [45,46,47]. MRSA isolates have historically been more resistant to antimicrobial treatments than MSSA bacteria because the SCCmec components of MRSA carry numerous resistance genes [48]. Similarly, MRSA isolates were more resistant to ampicillin, erythromycin, ciprofloxacin, and tetracycline than MSSA isolates in this study. These findings suggested that the colonization of multi-drug resistant isolates in pediatric patients may make further clinical treatment of highly virulent MRSA infections difficult.

### 3.3. Identification and Distribution of Virulent Factors

Virulent factors were compared to determine the pathogenicity in these *S. aureus* isolates. In total, 14 virulent factors were screened among 81 *S. aureus* strains including the functional factors of panton-valentine leucocidin, toxic shock syndrome toxin, staphylokinase, exfoliative toxin, and enterotoxin (Figure 3). Remarkably, *lu*kF-PV and *luk*S-PV were dominantly carried by CC22 (n = 23), CC59 (n = 4), CC338 (n = 2), CC25 (n = 2), CC88 (n = 2), and CC15 (n = 1) (Figure 3, Supplementary Table S2). However, *tsst*-1 genes were detected only in CC188 (n = 2), CC7 (n = 1), and CC15 (n = 1), while exfoliative toxin genes (*eta*, *etb*) were found only in CC121. The current isolates showed a lower prevalence of enterotoxin (24.7%, 20/81). 

Previous studies have reported the low carriage of CC398 virulent factors in Chinese adults [18]. In our study, CC398 carried only *sak* (n = 6) and *scn* (n = 5). However, our study illustrated the high carriage rate of Panton–Valentine leucocidin in CC22. The *pvl* gene might cause hematogenous osteomyelitis (OM) in both children and adults [1]. Previous studies reported that only 1.6% of MSSA fecal isolates from adults tested positive for *pvl* [38]. The overall detection rate of *pvl* in fecal sample isolates from pediatric patients in the United States is less than 10% [49]. However, the current high prevalence of *pvl* in pediatric patients implies a hypervirulent signature, and further research is needed on the clinical applicability of these CC types. 

The *scn* gene was observed in 34 of the present isolates including the types CC1 (n = 2), CC5 (n = 1), CC6 (n = 2), CC7 (n = 2), CC20 (n = 1), CC22 (n = 2), CC30 (n = 1), CC59 (n = 7), CC121 (n = 1), CC338 (n = 2), and CC1281 (n = 2) (Figure 3, Supplementary Table S2). The *scn* gene has been detected at high frequencies from human hosts as a marker of the immune evasion cluster (IEC), suggesting that the gene can be used to identify strains transmitted from animals, and environments to humans [50,51]. Adult patients typically have a higher prevalence of the *scn* gene. A previous study in China reported a prevalence of 82.8% of *scn* in fecal MSSA isolates from adult patients [18]. However, researchers reported that the *scn* gene was not detected in *S. aureus* isolated from the adenoid tissue of 112 children with adenoid symptoms [52]. In our study, only 41.98% of the isolates possessed the *scn* gene, indicating that the sources of infections in children are diverse, including both human-to-human transmission and environmental and nosocomial infections.

Generally, our study demonstrated that the frequencies of the staphylococcal enterotoxin *sea* (7.3%), *seb* (12.7%), *sec* (12.7%), *seh* (5.5%), *selk* (5.5%), *sell* (12.7%), and *selq* (5.5%) were low in the MSSA isolates compared with Chinese adults [18]. However, a previous Spanish study reported that none of the *S. aureus* strains in healthy human feces contained such an enterotoxin gene cluster [44]. In this study, most of the enterotoxin gene was dispersed in CC188 and CC59, suggesting that *S. aureus* infections in infants are still controlled by other virulent factors such as *luk*F-PV and that enterotoxin-related genes are not significant in these strains. 

## 4. Conclusions

In conclusion, comparative genomic analyses were enrolled for the molecular characteristics of methicillin-resistant and -susceptible *S. aureus* from pediatric patients in Suzhou, China. A total of 18 unique ST types, of which ST22 (28.4%) and ST59 (13.6%) were identified as the most typical strains. Most strains were from acute/chronic otitis externa(n = 23), otitis media(n = 11), and soft tissue infections (n = 9), of which 26 strains were identified as MRSA. The present study also observed an association between different CC types/ST and the age of pediatric patients and that CC398 was the predominant type in neonates under 1 month of age, while CC22 was mainly found in other infants. Additionally, 17 *S. aureus* isolates were resistant to at least three antimicrobials and the majority of them belonged to CC59 (n = 11), while numerous virulent factors were screened positive including the functional factors of Panton–Valentine leucocidin, toxic shock syndrome toxin, staphylokinase, exfoliative toxin, and enterotoxin. Together, the present study provided a phylogenetic and genotypic comparison of *S. aureus* from Chinese pediatric patients in Suzhou city. Our results suggested that the colonization of multi-drug resistant isolates may make further clinical concern among pediatric patients in eastern China.

## Figures and Tables

**Table 1:** CC, MRSA/MSSA, ST, spa types, and phenotypic antimicrobial resistance of *S. aureus* isolates from this study.

CCtype	MRSA/MSSA	ST ^a^	Spa Type ^b^	Phenotypic Antimicrobial Resistance ^c^
CC1	0/4	ST1 (3)	t127 (2), ND (1)	AMP (3), ERY (1), PEN (3), TET (1)
CC5	0/1	ST5 (1)	t1107 (1)	AMP (1), PEN (1)
CC6	1/1	ST6 (2)	t304 (1), t701 (1)	AMP (1), FOX (1), PEN (1), TET (1)
CC7	0/2	ST7 (2)	t91 (1), t19626 (1)	AMP (1), ERY (1), PEN (1)
CC8	0/1	ST630 (1)	t377 (1)	AMP (1), PEN (1)
CC15	0/5	ST15 (5)	t84 (1), t393 (1), t774 (1), t14014 (2)	AMP (4), ERY (1), PEN (4)
CC20	0/1	ST20 (1)	t14029 (1)	AMP (1), ERY (1), PEN (1)
CC22	8/15	ST22 (23)	t309 (17), t5763 (1), t13828 (1), ND (4)	AMP (22), ERY (9), FOX (4), PEN (22)
CC25	0/3	ST25 (3)	t78 (2), t280 (1)	AMP (3), FOX (1), PEN (3)
CC30	1/0	ST30 (1)	t19 (1)	ERY (1), FOX (1), PEN (1)
CC59	9/2	ST59 (11)	t437 (6), t441 (2), ND (3)	AMP (3), ERY (6), FOX (6), GEN (2), PEN (8), TET (1)
CC88	1/2	ST88 (3)	t2649 (1), t4780 (1), t10777 (1)	AMP (1), ERY (2), FOX (1), PEN (1)
CC121	1/4	ST121 (5)	t2092 (3), t1425 (1), t2019 (1), t4780 (1)	AMP (3), ERY (2), FOX (3), GEN (2), PEN (4)
CC188	0/5	ST188 (5)	t189 (4), t2883 (1)	AMP (5), ERY (5), FOX (4), GEN (4), PEN (5), TET (3)
CC338	2/0	ST338 (2)	t437 (2)	AMP (2), ERY (2), FOX (1), GEN (2), PEN (2), TET (1)
CC398	3/5	ST398 (8)	t34 (3), t571 (3), t1184 (1), t1456 (1)	AMP (6), CLI (2), ERY (5), FOX (3), GEN (2), PEN (6), TET (3)
CC1281	0/2	ST1281 (1)	t164 (1)	AMP (2), ERY (1), FOX (1), PEN (2)
		ST2631 (1)	t3277 (1)	
Not determined	0/3	Not determined (3)	t78 (1), t309 (1), t1376 (1)	AMP (1), ERY (1), PEN (1)

^a^ Numbers in parentheses are the number of isolates per sequence (ST) type. ^b^ Numbers in parentheses are the number of isolates per spa type. ND: not determined. ^c^ Numbers in parentheses are the number of isolates from each antimicrobial.

## Data Availability

The raw reads from this study are submitted to the China National GenBank under the link of https://bigd.big.ac.cn/gsa/browse/CRA009709, accessed on 7 February 2023.

## Supplementary Materials

The following supporting information can be downloaded at: www.mdpi.com/article/10.3390/pathogens12040549/s1, Table S1. Clinic data of pediatric patients; Table S2. Detail background on the range of ages and infections among the infants enrolled in this study.

## References

[1] de Araujo F.P., Monaco M., Del Grosso M., Pirolo M., Visca P., Pantosti A. (2021). Staphylococcus aureus clones causing osteomyelitis: A literature review (2000–2020). J. Glob. Antimicrob. Resist..

[2] McMullan B.J., Campbell A.J., Blyth C.C., McNeil J.C., Montgomery C.P., Tong S.Y., Bowen A.C. (2020). Clinical Management of *Staphylococcus aureus* Bacteremia in Neonates, Children, and Adolescents. Pediatrics.

[3] Rhee Y., Aroutcheva A., Hota B., Weinstein R.A., Popovich K.J. (2015). Evolving Epidemiology of *Staphylococcus aureus* Bacteremia. Infect. Control. Hosp. Epidemiol..

[4] Fu P., Xu H., Jing C., Deng J., Wang H., Hua C., Chen Y., Chen X., Zhang T., Zhang H. (2021). Bacterial Epidemiology and Antimicrobial Resistance Profiles in Children Reported by the ISPED Program in China, 2016 to 2020. Microbiol. Spectr..

[5] Al-Sadi A.M., Al-Oweisi F.A., Edwards S.G., Al-Nadabi H., Al-Fahdi A.M. (2015). Genetic analysis reveals diversity and genetic relationship among Trichoderma isolates from potting media, cultivated soil and uncultivated soil. BMC Microbiol..

[6] Klein E., Sun L., Smith D., Laxminarayan R. (2013). The Changing Epidemiology of Methicillin-Resistant Staphylococcus aureus in the United States: A National Observational Study. Am. J. Epidemiol..

[7] Sutter D.E., Milburn E., Chukwuma U., Dzialowy N., Maranich A.M., Hospenthal D.R. (2016). Changing Susceptibility of *Staphylococcus aureus* in a US Pediatric Population. Pediatrics.

[8] Vanderkoo F.D.O.G., Gregson F.D.B., Kellner J., Laupland F.K.B. (2011). Staphylococcus aureus bloodstream infections in children: A population-based assessment. Paediatr. Child Heal..

[9] Rojo P., Barrios M., Palacios A., Gomez C., Chaves F. (2010). Community-associated *Staphylococcus aureus* infections in children. Expert Rev. Anti-infective Ther..

[10] Pennone V., Prieto M., Álvarez-Ordóñez A., Cobo-Diaz J.F. (2022). Antimicrobial Resistance Genes Analysis of Publicly Available *Staphylococcus aureus* Genomes. Antibiotics.

[11] Ai X., Gao F., Yao S., Liang B., Mai J., Xiong Z., Chen X., Liang Z., Yang H., Ou Z. (2020). Prevalence, Characterization, and Drug Resistance of Staphylococcus Aureus in Feces From Pediatric Patients in Guangzhou, China. Front. Med..

[12] Costa A.R., Batistão D.W.F., Ribas R.M., Sousa A.M., Pereira M.O., Botelho C.M. (2013). Staphylococcus aureus virulence factors and disease. A. Mendez-Vilas, Microbial Pathogens and Strategies for Combating Them: Science, Technology and Education.

[13] Argudín M.Á., Mendoza M.C., Rodicio M.R. (2010). Food Poisoning and Staphylococcus aureus Enterotoxins. Toxins.

[14] Musser J.M., Schlievert P.M., Chow A.W., Ewan P., Kreiswirth B.N., Rosdahl V.T., Naidu A.S., Witte W., Selander R.K. (1990). A single clone of Staphylococcus aureus causes the majority of cases of toxic shock syndrome. Proc. Natl. Acad. Sci. USA.

[15] Özekinci T., Dal T., Yanık K., Özcan N., Can T.A., Yıldırım H.I., Kandemir I. (2014). Panton-Valentine leukocidin in community and hospital-acquiredStaphylococcus aureusstrains. Biotechnol. Biotechnol. Equip..

[16] Plano L.R. (2004). Staphylococcus aureus exfoliative toxins: How they cause disease. J. Investig. Dermatol..

[17] McCarthy A.J., Lindsay J.A. (2013). Staphylococcus aureus innate immune evasion is lineage-specific: A bioinfomatics study. Infect. Genet. Evol..

[18] Li Y., Tang Y., Jiang Z., Wang Z., Li Q., Jiao X. (2022). Molecular Characterization of Methicillin-Sensitive *Staphylococcus aureus* from the Intestinal Tracts of Adult Patients in China. Pathogens.

[19] Woods C.R., Bradley J.S., Chatterjee A., A Copley L., Robinson J., Kronman M.P., Arrieta A., Fowler S.L., Harrison C., A Carrillo-Marquez M. (2021). Clinical Practice Guideline by the Pediatric Infectious Diseases Society and the Infectious Diseases Society of America: 2021 Guideline on Diagnosis and Management of Acute Hematogenous Osteomyelitis in Pediatrics. J. Pediatr. Infect. Dis. Soc..

[20] Gurevich A., Saveliev V., Vyahhi N., Tesler G. (2013). QUAST: Quality assessment tool for genome assemblies. Bioinformatics.

[21] Zhou Z., Alikhan N.-F., Mohamed K., Fan Y., Achtman M., Brown D., Chattaway M., Dallman T., Delahay R., the Agama Study Group (2020). The EnteroBase user’s guide, with case studies on Salmonella transmissions, Yersinia pestis phylogeny, and Escherichia core genomic diversity. Genome Res..

[22] Larsen M.V., Cosentino S., Rasmussen S., Friis C., Hasman H., Marvig R.L., Jelsbak L., Sicheritz-Pontéen T., Ussery D.W., Aarestrup F.M. (2012). Multilocus Sequence Typing of Total-Genome-Sequenced Bacteria. J. Clin. Microbiol..

[23] Feil E.J., Li B.C., Aanensen D.M., Hanage W.P., Spratt B.G. (2004). eBURST: Inferring Patterns of Evolutionary Descent among Clusters of Related Bacterial Genotypes from Multilocus Sequence Typing Data. J. Bacteriol..

[24] Kaas R.S., Leekitcharoenphon P., Aarestrup F.M., Lund O. (2014). Solving the Problem of Comparing Whole Bacterial Genomes across Different Sequencing Platforms. PLoS ONE.

[25] Liu B., Zheng D., Jin Q., Chen L., Yang J. (2018). VFDB 2019: A comparative pathogenomic platform with an interactive web interface. Nucleic Acids Res..

[26] Johansson M.H.K., Bortolaia V., Tansirichaiya S., Aarestrup F.M., Roberts A.P., Petersen T.N. (2020). Detection of mobile genetic elements associated with antibiotic resistance in *Salmonella enterica* using a newly developed web tool: MobileElementFinder. J. Antimicrob. Chemother..

[27] Bortolaia V., Kaas R.S., Ruppe E., Roberts M.C., Schwarz S., Cattoir V., Philippon A., Allesoe R.L., Rebelo A.R., Florensa A.F. (2020). ResFinder 4.0 for predictions of phenotypes from genotypes. J. Antimicrob. Chemother..

[28] Zhou Z., Alikhan N.-F., Sergeant M.J., Luhmann N., Vaz C., Francisco A.P., Carriço J.A., Achtman M. (2018). GrapeTree: Visualization of core genomic relationships among 100,000 bacterial pathogens. Genome Res..

[29] Letunic I., Bork P. (2019). Interactive Tree Of Life (iTOL) v4: Recent updates and new developments. Nucleic Acids Res..

[30] Saini S., Gupta N., Sachdeva O.P., Aparna, Seema (2005). Bacteriological study of paediatric and adult chronic suppurative otitis media. Indian J. Pathol. Microbiol..

[31] Ding Y.L., Fu J., Chen J., Mo S.F., Xu S., Lin N., Qin P., McGrath E. (2018). Molecular characterization and antimicrobial susceptibility of Staphylococcus aureus isolated from children with acute otitis media in Liuzhou, China. BMC Pediatr..

[32] Alter S.J., Vidwan N.K., Sobande P.O., Omoloja A., Bennett J.S. (2011). Common Childhood Bacterial Infections. Curr. Probl. Pediatr. Adolesc. Heal. Care.

[33] Da Silva A.G., Baines S., Carter G.P., Heffernan H., French N.P., Ren X., Seemann T., Bulach D., Kwong J., Stinear T.P. (2017). A phylogenomic framework for assessing the global emergence and evolution of clonal complex 398 methicillin-resistant Staphylococcus aureus. Microb. Genom..

[34] Aires-De-Sousa M. (2017). Methicillin-resistant Staphylococcus aureus among animals: Current overview. Clin. Microbiol. Infect..

[35] Li S., Sun S., Yang C., Chen H., Yin Y., Li H., Zhao C., Wang H. (2018). The Changing Pattern of Population Structure of Staphylococcus aureus from Bacteremia in China from 2013 to 2016: ST239-030-MRSA Replaced by ST59-t437. Front. Microbiol..

[36] He C., Xu S., Zhao H., Hu F., Xu X., Jin S., Yang H., Gong F., Liu Q. (2018). Leukotoxin and pyrogenic toxin Superantigen gene backgrounds in bloodstream and wound Staphylococcus aureus isolates from eastern region of China. BMC Infect. Dis..

[37] Liu C., Chen Z.-J., Sun Z., Feng X., Zou M., Cao W., Wang S., Zeng J., Wang Y., Sun M. (2015). Molecular characteristics and virulence factors in methicillin-susceptible, resistant, and heterogeneous vancomycin-intermediate Staphylococcus aureus from central-southern China. J. Microbiol. Immunol. Infect..

[38] Wang B., Xu Y., Zhao H., Wang X., Rao L., Guo Y., Yi X., Hu L., Chen S., Han L. (2022). Methicillin-resistant *Staphylococcus aureus* in China: A multicentre longitudinal study and whole-genome sequencing. Emerg. Microbes Infect..

[39] Ladhani S.N., Lucidarme J., Newbold L.S., Gray S.J., Carr A.D., Findlow J., Ramsay M.E., Kaczmarski E.B., Borrow R. (2012). Invasive Meningococcal Capsular Group Y Disease, England and Wales, 2007–2009. Emerg. Infect. Dis..

[40] Dong Q., Liu Y., Li W., Chen M., Li W., Wang X., Fu J., Ye X. (2020). Phenotypic and Molecular Characteristics of Community-Associated Staphylococcus aureus Infection in Neonates. Infect. Drug Resist..

[41] Chen Y., Sun L., Ba X., Jiang S., Zhuang H., Zhu F., Wang H., Lan P., Shi Q., Wang Z. (2021). Epidemiology, evolution and cryptic susceptibility of methicillin-resistant Staphylococcus aureus in China: A whole-genome-based survey. Clin. Microbiol. Infect..

[42] Xu Z., Xie J., Peters B.M., Li B., Li L., Yu G., Shirtliff M.E. (2017). Longitudinal surveillance on antibiogram of important Gram-positive pathogens in Southern China, 2001 to 2015. Microb. Pathog..

[43] Bush K., Bradford P.A. (2020). Epidemiology of β-Lactamase-Producing Pathogens. Clin. Microbiol. Rev..

[44] Benito D., Lozano C., Jiménez E., Albújar M., Gómez A., Rodríguez J., Torres C. (2015). Characterization of Staphylococcus aureus strains isolated from faeces of healthy neonates and potential mother-to-infant microbial transmission through breastfeeding. FEMS Microbiol. Ecol..

[45] Jian Y., Lv H., Liu J., Huang Q., Liu Y., Liu Q., Li M. (2020). Dynamic Changes of Staphylococcus aureus Susceptibility to Vancomycin, Teicoplanin, and Linezolid in a Central Teaching Hospital in Shanghai, China, 2008–2018. Front. Microbiol..

[46] Saravolatz L.D., Pawlak J. (2023). In vitro activity of fosfomycin alone and in combination against Staphylococcus aureus with re-duced susceptibility or resistance to methicillin, vancomycin, daptomycin or linezolid. J. Antimicrob. Chemo-Ther..

[47] Kadkhoda H., Ghalavand Z., Nikmanesh B., Kodori M., Houri H., Maleki D.T., Bavandpour A.K., Eslami G. (2020). Characterization of biofilm formation and virulence factors of Staphylococcus aureus isolates from paediatric patients in Tehran, Iran. Iran. J. Basic Med. Sci..

[48] Pistiki A., Monecke S., Shen H., Ryabchykov O., Bocklitz T.W., Rösch P., Ehricht R., Popp J. (2022). Comparison of Different Label-Free Raman Spectroscopy Approaches for the Discrimination of Clinical MRSA and MSSA Isolates. Microbiol. Spectr..

[49] Khamash D.F., Voskertchian A., Tamma P.D., Akinboyo I.C., Carroll K.C., Milstone A.M. (2018). Increasing Clindamycin and Trimethoprim-Sulfamethoxazole Resistance in Pediatric Staphylococcus aureus Infections. J. Pediatr. Infect. Dis. Soc..

[50] Li H., Andersen P.S., Stegger M., Sieber R.N., Ingmer H., Staubrand N., Dalsgaard A., Leisner J.J. (2019). Antimicrobial Resistance and Virulence Gene Profiles of Methicillin-Resistant and -Susceptible Staphylococcus aureus From Food Products in Denmark. Front. Microbiol..

[51] Abdullahi I.N., Lozano C., Saidenberg A.B.S., Latorre-Fernández J., Zarazaga M., Torres C. (2023). Comparative review of the nasal carriage and genetic characteristics of Staphylococcus aureus in healthy livestock: Insight into zoonotic and anthroponotic clones. Infect. Genet. Evol..

[52] Ziasistani M., Dabiri S., Abadi M.F.S., Afshari S.A.K., Ghaioumy R., Morones-Ramírez J.R., Tabatabaeifar F., Kalantar-Neyestanaki D. (2020). Determination of antibiotic resistance genes, immune evasion cluster and agr types among Staphylococcus aureus strains isolated from children with adenoiditis. Gene Rep..

